# Real-World Outcomes of [^177^Lu]Lu-PSMA-617 in the French Early-Access Program for Patients with Metastatic Castration-Resistant Prostate Cancer

**DOI:** 10.2967/jnumed.126.272721

**Published:** 2026-10

**Authors:** Clément Bailly, Lavinia Vija, François Somme, Vincent Massard, Philippe Barthélémy, Anne-Laure Giraudet, Yann Godbert, Aude Fléchon, Sylvie Girault, Sophie Abadie-Lacourtoisie, Caroline Viala, Alix Douillet, Stephanie Chêne, Pierre Olivier, Guilhem Roubaud

**Affiliations:** 1 Nuclear Medicine Department, Nantes University Hospital, Nantes, France;; 2 Department of Nuclear Medecine, Oncopole Claudius Regaud, Toulouse, France;; 3 Nuclear Medicine and Molecular Imaging, Institut Strauss, Strasbourg, France;; 4 Medical Oncology Department, Institut de Cancérologie de Lorraine, Vandoeuvre-lès-Nancy, France;; 5 Department of Medical Oncology, University Hospital Strasbourg, Strasbourg, France;; 6 Lumen Nuclear Medicine Department, Centre Léon Bérard, Lyon, France;; 7 Department of Thyroid Oncology and Nuclear Medicine, Institut Bergonié, Bordeaux, France;; 8 Department of Medical Oncology, Urological Oncology Centre Léon Bérard, Lyon, France;; 9 Department of Nuclear Medicine, ICO Pays de la Loire, Angers, France;; 10 ICO Paul Papin, Angers, France;; 11 Nantes University Hospital, Nantes, France;; 12 Medical Department, Novartis, Rueil-Malmaison, France;; 13 Department of Nuclear Medicine, CHRU Nancy, Nancyclotep Imaging Platform, Nancy, France; and; 14 Department of Medical Oncology, Institut Bergonié, Bordeaux, France

**Keywords:** [^177^Lu]Lu-PSMA-617, mCRPC, early-access program, real-world evidence

## Abstract

Metastatic castration-resistant prostate cancer (mCRPC) remains challenging to treat, especially after failure of standard therapies, such as androgen receptor pathway inhibitors and taxane-based chemotherapy. Radiopharmaceutical therapy with [^177^Lu]Lu-PSMA-617 targets prostate-specific membrane antigen (PSMA)–positive tumor cells. In the VISION trial, [^177^Lu]Lu-PSMA-617 has demonstrated significant benefits in terms of median overall survival and a significant improvement of median radiographic progression-free survival (PFS) versus standard of care. Recent guidelines support its use in PSMA-positive mCRPC. In France, [^177^Lu]Lu-PSMA-617 was available via an early-access program from December 1, 2021, to April 29, 2025. This analysis reports the largest real-world cohort of patients with mCRPC treated with [^177^Lu]Lu-PSMA-617, evaluating its use, safety, and effectiveness in routine clinical practice. **Methods:** This multicenter descriptive cohort included adult patients with mCRPC who had progressive disease after at least 1 androgen receptor pathway inhibitor and 1 taxane-based treatment, and 1 positive PSMA PET scan. Patients received up to 6 cycles of intravenous [^177^Lu]Lu-PSMA-617 every 6 wk. Clinical evaluation, prostate-specific antigen (PSA) status, imaging assessments, and treatment-related adverse events were prospectively collected during regular follow-up. PFS was analyzed using Kaplan–Meier methods. Outcomes were compared descriptively to the VISION trial. **Results:** From December 2021 to April 2025, 3709 patients from 46 centers received at least 1 dose and 2476 had more than 8 mo of follow-up for efficacy analysis. Compared with VISION, patients were older, more heavily pretreated, and had more lymph node metastases but lower baseline PSA. Median imaging PFS was 7.6 mo versus 8.7 mo in VISION. Disease control was achieved in 82% according to PSA criteria. Some patients with early PSA progression after the first cycle subsequently showed a response, suggesting a flare-up effect. The safety profile was manageable, with treatment discontinuations mainly due to progression. No new safety signals were identified. Subgroup analyses confirmed efficacy in elderly patients (aged >75 y) and more favorable outcomes in patients less heavily pretreated with taxane-based chemotherapy. **Conclusion:** The French early-access program confirms a favorable benefit–risk profile for [^177^Lu]Lu-PSMA-617 in a real-world mCRPC population. Use trends suggest earlier use of the treatment after 1 taxane-based treatment, with patients being less pretreated and characterized by fewer metastases.

## Visual Abstract

**Figure:**
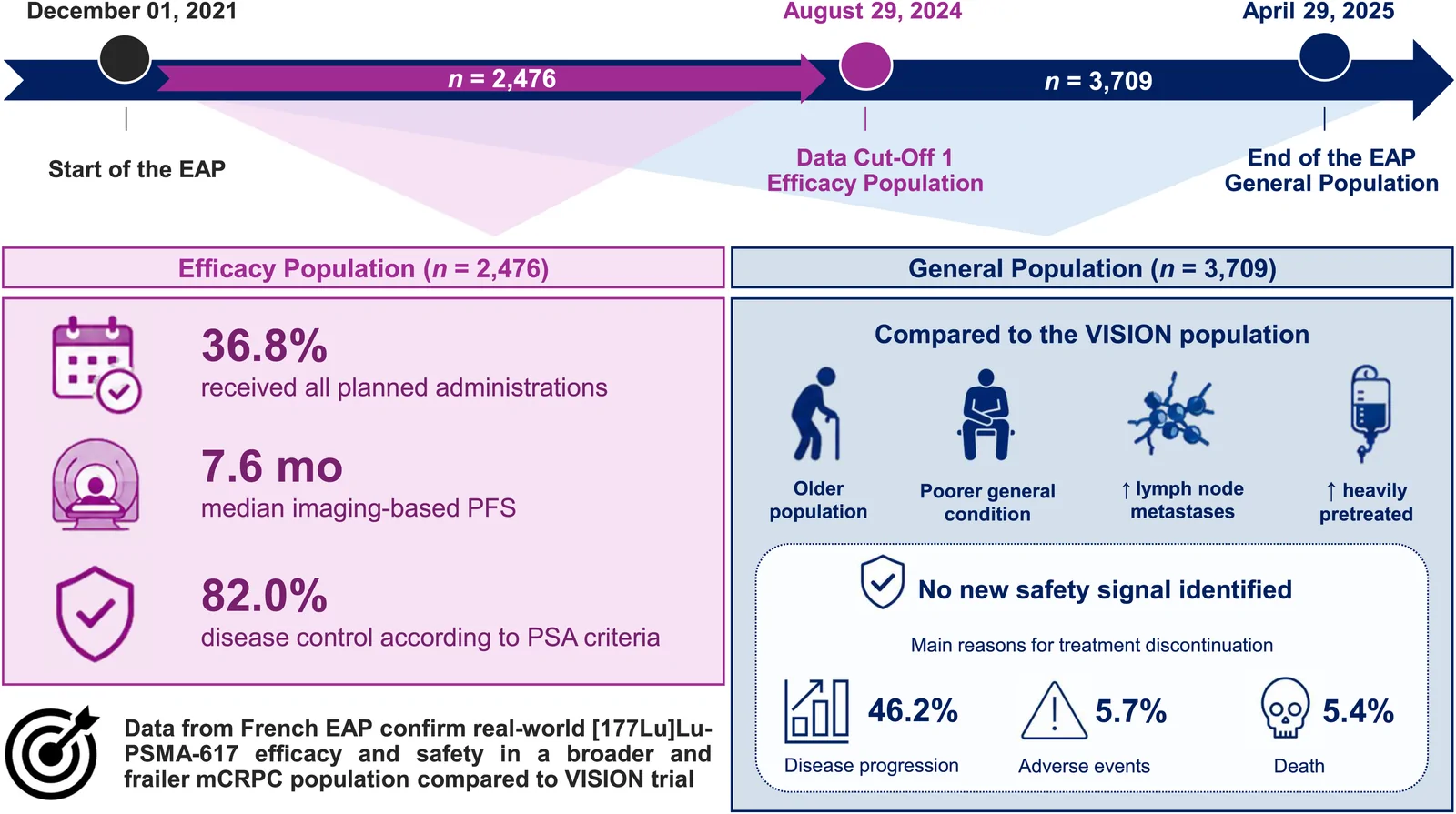

The management of metastatic castration-resistant prostate cancer (mCRPC) continues to present significant challenges, particularly for patients who have progressed after receiving standard therapies, such as androgen receptor pathway inhibitor (ARPI) and taxane-based chemotherapy. Radiopharmaceutical therapy targeting prostate-specific membrane antigen (PSMA) has emerged as a novel treatment modality for these patients, with [^177^Lu]Lu-PSMA-617 being the first radiopharmaceutical therapy approved for mCRPC. This therapy binds with high affinity and specificity to PSMA to deliver targeted β-particle radiation to PSMA-positive lesions, providing a new treatment option for patients with advanced prostate cancer (1).

The VISION phase 3 trial demonstrated that [^177^Lu]Lu-PSMA-617 in combination with standard of care (e.g., with or without a concomitant ARPI) significantly improved radiographic progression-free survival (PFS) and overall survival compared with standard of care alone, in patients with PSMA-positive mCRPC who had previously received at least 1 ARPI and 1 taxane-based chemotherapy (1). The phase 2 TheraP trial also demonstrated the efficacy of [^177^Lu]Lu-PSMA-617 and its favorable safety profile (2). These data support the use of [^177^Lu]Lu-PSMA-617 as an alternative to cabazitaxel in patients with mCRPC previously treated with at least 1 ARPI and 1 taxane-based chemotherapy. Given these outcomes, guidelines from the French Association of Urology, the European Society for Medical Oncology, and the European Association of Nuclear Medicine recommend [^177^Lu]Lu-PSMA-617 as a treatment option for patients with advanced PSMA-expressing prostate cancer who have received at least 1 ARPI and 1 taxane-based chemotherapy (3–5) and even after only 1 ARPI (6).

Although the VISION and TheraP trials provide robust evidence in a selected population, they also highlight the need for complementary real-world data to understand the use and outcomes of [^177^Lu]Lu-PSMA-617 therapy in routine clinical practice among broader and more diverse patient populations.

In France, [^177^Lu]Lu-PSMA-617 was available through an early-access program (EAP) that began on December 1, 2021, and ended on April 29, 2025. We report here data from patients included in this EAP which represents the largest real-world cohort of patients with mCRPC treated with this therapy worldwide. This comprehensive dataset provides the opportunity to evaluate the safety and efficacy of [^177^Lu]Lu-PSMA-617 in routine clinical practice.

## MATERIALS AND METHODS

This multicenter cohort study was conducted within the French EAP for [^177^Lu]Lu-PSMA-617. Eligible patients were adult men with mCRPC progressing after at least 1 ARPI and 1 taxane-based chemotherapy regimen, and positive PSMA PET imaging. Patients received up to 6 cycles of [^177^Lu]Lu-PSMA-617 (7.4 GBq ± 10% every 6 wk).

Efficacy endpoints included prostate-specific antigen (PSA), clinical, and imaging responses categorized as improvement, stabilization, or progression according to investigator assessment. Safety analyses included treatment-related adverse events (AEs).

Descriptive statistics summarize baseline characteristics and outcomes. PFS was estimated using the Kaplan–Meier method, and exploratory subgroup analyses were performed using Mann–Whitney tests. Full methodologic details are provided in the supplemental materials (available at http://jnm.snmjournals.org).

## RESULTS

### Patient Population

Overall, 3709 patients from 46 centers across France were enrolled in the EAP and received at least 1 dose of [^177^Lu]Lu-PSMA-617. Participating centers were located across France, with an additional center in Monaco. Participating sites included 24 university hospitals (52.2%), 17 cancer centers (37.0%), and 5 community hospitals (10.9%). A complete list of participating centers and investigators is provided in Supplemental Table 1.

Baseline patient and treatment characteristics, concomitant medications, and safety data were described within the overall cohort (*n* = 3709) based on data available up to April 29, 2025. Efficacy analyses were conducted in a predefined population of 2476 patients who received their first injection before the efficacy cutoff date of August 31, 2024.

### Baseline Characteristics

Patients enrolled in the French EAP had a median age of 73.8 y, with 5.4% aged over 85 y. Overall, 87% presented with a good Eastern Cooperative Oncology Group Performance Status (ECOG-PS) of 0–1. Metastatic sites included bone (92.1%), lymph nodes (59.5%), lung (8.2%), liver (8.3%), and brain (1.2%). Detailed baseline characteristics for the overall cohort are presented in Table 1.

**TABLE 1.** Baseline Characteristics of EAP Population (*n* = 3709)

Characteristic	Value
Age (y)	
Median	73.8 (37–100)
≥75	1602 (43.2)
≥85	199 (5.4)
ECOG-PS	
Missing data	2
0–1	3223 (87.0)
2	465 (12.5)
3	18 (0.5)
4	1 (0.0)
Site of disease	
Bone	3417 (92.1)
Lymph node	2208 (59.5)
Liver	307 (8.3)
Lung	305 (8.2)
Brain	43 (1.2)
Bone only	1234 (33.3)
Bone + lymph node	1382 (37.2)
Bone + lymph node + lung	146 (3.9)
Bone + lymph node + liver	135 (3.6)
Bone + lymph node + others	178 (4.8)
PSA level (ng/mL)	
Median	41.6 (0–6972)
100% of PSMA-positive lesions	
Yes	2641 (71.2)
Time between TEP-PSMA and inclusion (mo)	
Median	0.4 (0.0–14.5)
Creatinine clearance	
Missing data	31
≥60	3338 (90.8)
30–60	334 (9.1)
<30	6 (0.2)

Data are number and percentage; continuous data are minimum and maximum in parentheses.

### Treatment History

The median number of prior systemic therapies received was 3, excluding androgen-deprivation therapy and novel drug classes under investigation. Over half of the patients (51.2%) had received at least 2 ARPIs, and 41.3% received at least 2 taxane-based chemotherapies. A total of 103 patients in the EAP cohort had previously received radiopharmaceutical treatments, including 98 (2.6% among the overall cohort) with ^223^Ra.

### Therapeutic Sequence

Patients in the EAP efficacy population had a median follow-up of 5.1 mo and a median of 4 treatment cycles. The median cumulative dose administered was 32.6 GBq. The number of cycles received according to the therapeutic sequence is described in Table 2. Patients who had received only 1 prior chemotherapy before [^177^Lu]Lu-PSMA-617 presented a higher median number of administered injections (5 vs. 4) and were more likely to complete all 6 planned cycles (45.0% vs. 36.5%).

**TABLE 2.** Previous Therapies and [^177^Lu]Lu-PSMA-617 Treatment

Parameter	1 chemotherapy + 1 ARPI*	1 chemotherapy + 2 ARPI^†^	2 chemotherapies + 1 ARPI^‡^	2 chemotherapies + 2 ARPIs^§^
Median number of [^177^Lu]Lu-PSMA-617 cycles	5	5	4	4
Interquartile range	3–6	3–6	2–6	2–6
Minimum, maximum	1, 6	1, 6	1, 6	1, 6
No. of patients who received the 6 doses	256 (43.2%)	187 (47.7%)	126 (34.7%)	246 (37.5%)

* *n* = 592.

† *n* = 392.

‡ *n* = 363.

§ *n* = 656.

Variations in sample sizes across analyses reflect expected differences in data completeness.

### Evolution of Patient Profiles over Time

Patient characteristics and treatment history by year of first [^177^Lu]Lu-PSMA-617 injection are summarized in Supplemental Table 2. This table summarizes baseline demographics and prior therapies over time.

### Concomitant Treatments

In the EAP cohort, 3296 patients (88.9%) received at least 1 of those 3 therapies during [^177^Lu]Lu-PSMA-617 treatment: androgen-deprivation therapy (97%), ARPI (24.4%), or bone resorptive agents (32.5%).

### Efficacy

Efficacy outcomes are summarized in Table 3 for the efficacy population (*n* = 2476), in which the observed median follow-up was 6.6 mo. The reported efficacy results correspond to the best response achieved by patients during the follow-up period. Disease control, defined as the proportion of patients achieving either stabilization or improvement of the disease, was observed in 82.2% of patients based on biologic criteria (PSA level), 95.3% according to clinical criteria, and 65.1% according to imaging criteria. The imaging-based PFS was 7.6 mo (95% CI, 7.2–7.7 mo) reported with the corresponding Kaplan–Meier curve in Figure 1.

**TABLE 3.** Efficacy Outcomes in EAP Cohort

Parameter	*n*
Best biologic response	
Missing data	139
PSA decrease	1632 (69.8%)
PSA stabilization	288 (12.3%)
PSA progression	401 (17.2%)
Not evaluable	16 (0.7%)
Best clinical response	
Missing data	83
Improvement	1160 (48.5%)
Stabilization	1120 (46.8%)
Progression	113 (4.7%)
Not evaluable	—
Best radiologic response	
Missing data	408
Improvement	870 (41.0%)
Stabilization	477 (23.1%)
Progression	682 (33.0%)
Not evaluable	39 (1.9%)

**FIGURE 1.**
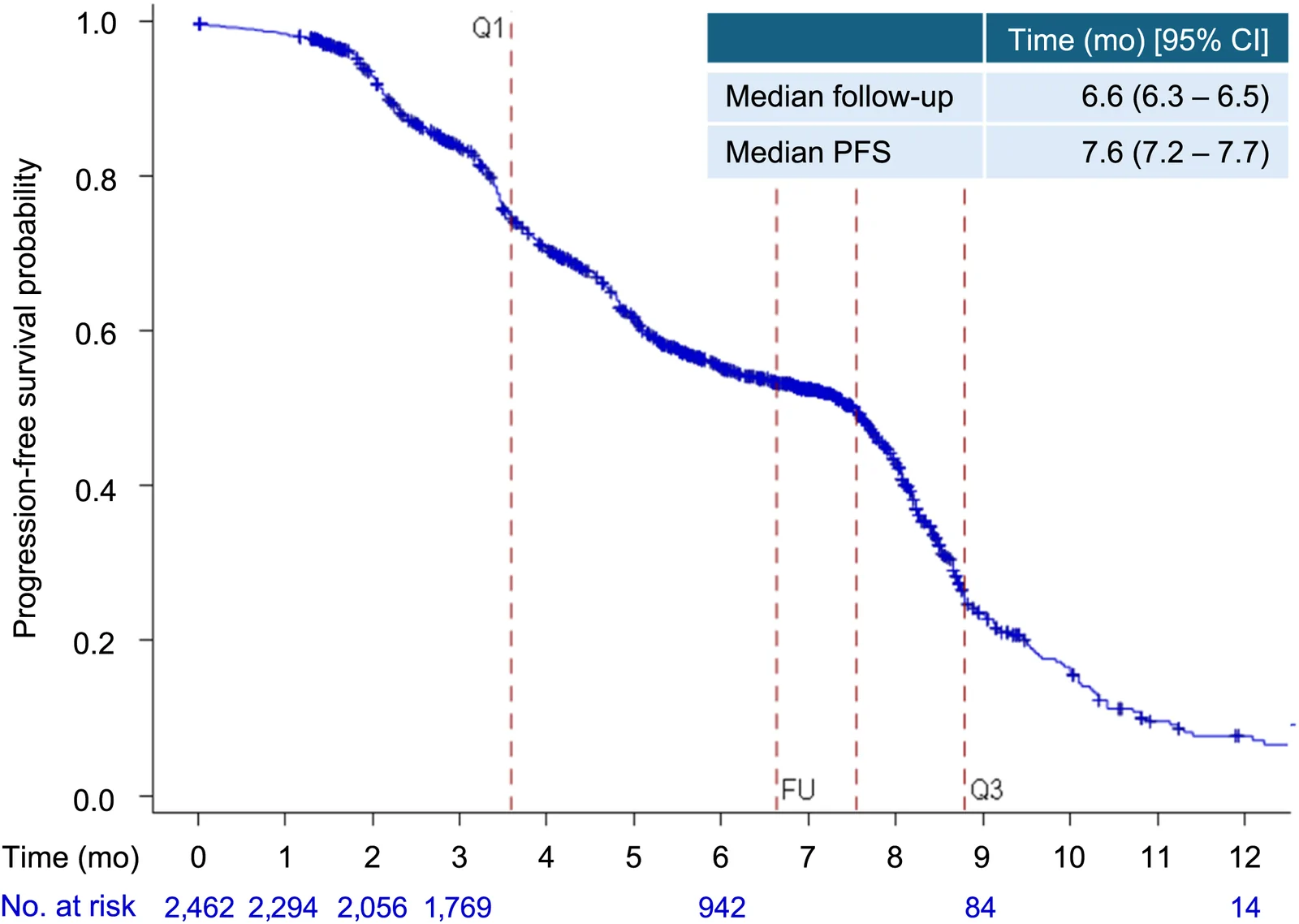
Imaging-based PFS among patients from efficacy population who received [^177^Lu]Lu-PSMA-617. Imaging-based PFS was defined as time from treatment initiation to imaging-documented disease progression. Disease progression was assessed according to local clinical practice at participating centers. Tick marks indicate censored observations. The median imaging-based PFS was 7.6 mo (95% CI, 7.2–7.7 mo).

Among the efficacy population, 596 (24.1%) showed initial disease progression based on PSA assessment after the first cycle. Of these, 54 subsequently showed stabilization (9.4%) and 312 showed a PSA decrease (54.1%) in later cycles, consistent with a flare-up effect. In contrast, 209 patients (36.2%) continued to meet the criteria for progression at cycle 2 or later assessments. Compared with the 312 responders, these 209 patients with continued progression seemed to have higher baseline PSA (54.0 ng/mL vs. 44.7 ng/mL) and lower PSMA positivity (68.8% vs. 81.4%). Most of them discontinued treatment earlier because of progression (83.7% vs. 27.3%). Imaging-based PFS was significantly shorter in this group (3.2 mo vs. 8.2 mo; *P* < 0.0001).

### Safety Data

At the data cutoff, 2435 patients had completed an end-of-treatment form. Of these, 36.8% (895/2435) received all planned treatment administrations, whereas 1540 patients experienced early interruption of treatment (63.2%). Early discontinuation included disease progression (46.2%), AEs (5.7%), death regardless of its relationship to treatment (5.4%), and other reasons (6.0%). On average, treatment discontinuation because of progression and death occurred 4.5 mo and 3.1 mo after inclusion, respectively.

By the data cutoff, a total of 462 patients (12.5%, 462/3709) reported at least 1 treatment-related AE, with 338 experiencing serious AEs and 12 fatal outcomes. Investigators documented 908 treatment-related AEs, including 598 classified as serious and 27 resulting in death. The distribution of AEs by standard of care is presented in Supplemental Table 3. Blood and lymphatic disorders accounted for the largest proportion, representing 48.5% of all AEs and 63.4% of serious AEs. The most frequently observed events in this category were thrombocytopenia (36.8%, 162/440), anemia (30.7%, 135/440), neutropenia (13.4%, 59/440), and leukopenia (9.3%, 41/440). The investigations’ standard of care constituted 23.0% of total AEs and 11.9% of serious AEs, with most events reflecting laboratory findings consistent with hematologic toxicity, including decreased hemoglobin (21.1%, 47/209), platelet count (17.2%, 36/209), and white blood cell count (7.7%, 16/209).

### Subanalyses

Preliminary subanalyses explored the impact of age (Supplemental Tables 4A–4G), predictive factors of clinical–biological response (Supplemental Tables 5A–5G), prior chemotherapy exposure (Supplemental Tables 6A–6G), concomitant ARPI use (Supplemental Tables 7A–7G), and renal impairment (Supplemental Tables 8A–8G).

Compared with patients 75 y old or younger, elderly patients had a poorer general condition (ECOG-PS 0–1, 84.1% vs. 89.0%), higher PSA levels (48.1 ng/mL vs. 36.0 ng/mL), and lower creatinine clearance (40.8% vs. 59.2%). Elderly patients were more likely to have received at least 1 prior ARPI (56.2% vs. 47.4%). Elderly patients completed all planned cycles slightly more often (42.8% vs. 38.7%), whereas discontinuation because of AEs was more frequent (8.0% vs. 4.3%). Median PFS was significantly higher in the elderly patients based on imaging (8.0 mo vs. 6.7 mo; *P* < 0.0001) and clinical examination (8.2 mo vs. 7.9 mo; *P* = 0.0005).

Overall, 1172 (31.6%) patients were classified as responders (decrease in PSA level and clinical improvement) and 2537 (68.4%) as nonresponders (PSA progression or clinical deterioration, and patients remaining stable or not fulfilling the response criteria). Responders had higher median baseline PSA level (50.9 ng/mL vs. 38.5 ng/mL) and were less likely to have hepatic or lung metastases (7.9% vs. 8.4% and 7.8% vs. 8.4%, respectively). There was a higher proportion of responders with 100% PSMA-positive lesions (82.5% vs. 66.1%). Responders more frequently received concomitant treatment (96.1% vs. 87.5%), especially ARPI (30.7% vs. 22.1%) and bone resorptive agents (41.9% vs. 30.9%).

Patients previously treated with 2 prior taxane regimens progressed more rapidly. In imaging-based evaluation, the median PFS was 7.7 mo after 1 regimen versus 6.8 mo after 2 (*P* = 0.0115). Clinical evaluation yielded a median PFS of 8.4 mo for 1 regimen versus 7.5 mo for 2 (*P* < 0.0001). PSA-based evaluation showed a median PFS of 4.7 mo in the 1-regimen group and 3.5 mo in the 2-regimen group (*P* = 0.0003). At baseline, patients pretreated with 2 taxane regimens had higher median PSA level (73.5 ng/mL vs. 28.4 ng/mL) and received more frequently multiple ARPIs (66.6% vs. 40.2%). Conversely, no significant differences in clinical, biological, or imaging-based PFS were observed between patients who had received 1 versus 2 ARPIs before treatment with [^177^Lu]Lu-PSMA-617.

Patients receiving concomitant ARPI tended to have a higher baseline PSA level (43.4 ng/mL vs. 33.0 ng/mL). They were more likely to have been pretreated with several ARPIs (59.8% vs. 48.8%) but received less frequently 2 previous taxane-based chemotherapies (37.8% vs. 42.3%). Patients who received a concomitant ARPI completed all planned cycles more often (45.7% vs. 34.4%). They also demonstrated slower progression compared with those without ARPI exposure. Median imaging-based PFS was 7.9 mo in the ARPI group versus 7.2 mo in the non-ARPI group (*P* < 0.0001). Similarly, median PFS by clinical assessment reached 8.4 mo with concomitant ARPI, compared with 8.0 mo without (*P* < 0.0001). PSA-based evaluation showed a median PFS of 4.9 mo with ARPI versus 3.8 mo without ARPI (*P* = 0.0041).

Overall, 340 patients had renal impairment (creatinine clearance of <60 mL/min). These patients were older (77.7 y vs. 73.5 y) and had higher baseline PSA levels (53.5 ng/mL vs. 40.2 ng/mL) compared with patients with normal renal function. Although differences were observed, no significant variation in imaging or clinical PFS was found between the groups. However, PSA response was higher in patients with creatinine clearance less than 60 mL/min compared with others (5.0 mo vs. 3.6 mo; *P* = 0.0012). Fewer patients with renal impairment completed all planned cycles (33.2% vs. 37.1%), and discontinuation because of AEs was more common in those patients compared with others (10.9% vs. 5.3%).

At data closure, 134 patients (3.6%) from the EAP received a second schedule of [^177^Lu]Lu-PSMA-617, as a retreatment period, and 4 patients received a third schedule (0.1%). The median number of cycles received was 3 during the retreatment phase. One patient received as many as 14 cycles with a cumulative administered activity of 99.04 GBq. No new safety signals or tolerability issues were identified during retreatment. This subpopulation will be the subject of a forthcoming detailed publication.

## DISCUSSION

This multicenter, real-world analysis provides important insights into the use of [^177^Lu]Lu-PSMA-617 for patients with mCRPC within the French EAP. To our knowledge, this is the largest published cohort in this setting. Our findings confirm the clinical benefit and manageable safety profile of [^177^Lu]Lu-PSMA-617 observed in the phase 3 VISION study, while also highlighting new considerations relevant to routine clinical practice (1).

### Contextualization in Relation to the VISION Study

The patient population in the EAP cohort was notably older (+3.8 y) with a higher proportion of patients aged 85 y or older (+3.9%). EAP patients were also in a poorer general condition, with a lower proportion of patients with good performance status (ECOG-PS 0–1) (−5.6%). Lymph node metastases were more prevalent (+9.8%). These data reflect the broader eligibility criteria in this EAP compared with VISION trial. Moreover, these patients were more heavily pretreated, with a higher proportion of patients having received at least 2 ARPIs (+12.5%) and 2 taxane-based chemotherapy regimens (+1.4%) before [^177^Lu]Lu-PSMA-617, compared with VISION. However, efficacy outcomes remain consistent with expectations based on the VISION trial. Indeed, patients from the EAP cohort received a median of 4 cycles of [^177^Lu]Lu-PSMA-617, and the median imaging-based PFS was 7.6 mo, compared with a median of 5 cycles and imaging-based PFS of 8.7 mo in the VISION trial. Toxicities were consistent between EAP and VISION cohorts, and no new safety signals were identified.

Importantly, a flare-up effect characterized by a transient increase in PSA or imaging findings shortly after treatment initiation was observed in a subset of patients. Indeed, among the 596 patients with initial PSA progression after the first cycle, over half later experienced a PSA decrease or stabilization. Median PSA-based PFS was 8.2, 5.2, and 3.2 mo for patients experiencing a PSA decrease, stabilization, or progression after the first treatment cycle, respectively. PSA responses in these patients suggest that an initial PSA rise does not necessarily indicate treatment failure. The flare-up effect has already been described in patients with mCRPC treated by [^177^Lu]Lu-PSMA-617 as well as with other treatments (7–9). This phenomenon is a temporary worsening of imaging findings or transient PSA rise occurring shortly after treatment initiation, potentially caused by bone healing and inflammatory responses rather than true disease progression. It usually resolves within weeks (10). Awareness of this phenomenon is essential to avoid misinterpretation of early progression and unnecessary discontinuation of effective therapy. Thus, disease progression should not be assessed using only PSA response between the first and second cycles.

Similar efficacy and safety outcomes have been reported in the U.S. expanded-access program and in Germany’s multicenter real-world cohorts, supporting the effectiveness and tolerability of [^177^Lu]Lu-PSMA-617 beyond highly selected clinical trial populations (11–13). Altogether, these findings confirm that [^177^Lu]Lu-PSMA-617 remains effective and well tolerated in routine clinical practice.

### Evolution of Patient Profiles

Our real-world data highlight a temporal shift in the selection of patients for [^177^Lu]Lu-PSMA-617 therapy within the French EAP. Over the course of the program, there was a progressive reduction in the proportion of patients with heavy prior exposure to systemic therapies (chemotherapy and ARPIs) as well as in the presence of bone, lymph node, liver, and lung metastases at baseline. Baseline PSA levels also declined steadily. These trends suggest that, with growing clinical experience, increased confidence in the therapy, and wider treatment availability, clinicians have gradually expanded access to [^177^Lu]Lu-PSMA-617 to patients with less advanced or less aggressive disease. This evolution highlights a shift toward earlier use of [^177^Lu]Lu-PSMA-617 in real-world practice.

Beyond the observed changes in baseline characteristics over time, our findings highlight an important temporal dynamic in the use of [^177^Lu]Lu-PSMA-617 within the French EAP. Initially, the treatment was predominantly reserved for heavily pretreated patients with advanced disease, reflecting cautious clinician adoption. This evolution likely reflects growing physician confidence in the treatment and its anticipated integration into earlier treatment lines. Recent evidence further supports the earlier use of PSMA-targeted radiopharmaceutical therapy. The PSMAfore trial has demonstrated efficacy and safety of [^177^Lu]Lu-PSMA-617 in taxane-naïve patients with mCRPC (14). In parallel, the PSMAddition study has reported its primary results in patients with metastatic hormone-sensitive prostate cancer not previously treated by chemotherapy (15).

Early data from the ENZA-p phase 2 trial reported potential overall survival and quality-of-life benefits with [^177^Lu]Lu-PSMA-617 plus enzalutamide (16). Similarly, a secondary analysis of VISION also suggests improved outcomes with concomitant ARPI use (17).

### Subanalyses

Several subanalyses have been conducted to further refine our understanding of [^177^Lu]Lu-PSMA-617 in real-world practice. Early results suggest that elderly patients show comparable benefit from treatment despite slightly higher AE-related discontinuation rates. Patients previously treated with 2 taxane regimens progressed faster than those receiving only 1. Concomitant ARPI use was associated with slower disease progression and greater treatment completion. Additional analyses are examining predictors of response, including disease characteristics and concomitant therapies, with the aim of refining patient selection. Finally, preliminary data on retreatment with [^177^Lu]Lu-PSMA-617 suggest relevant efficacy and an acceptable safety profile. These ongoing investigations are expected to provide valuable insights for optimizing therapeutic strategies and personalizing treatments in this population. Patients with renal impairment were older and had higher disease markers. Their treatment response appeared similar to others, but some discontinued treatment more often because of AEs. Future studies are planned to focus on patients with renal impairment, aiming to better understand the treatment’s efficacy and safety in this specific subgroup (NCT06004661).

### Strengths and Limitations

This study has several notable strengths. To our knowledge, this is the largest real-world cohort of patients with mCRPC treated with [^177^Lu]Lu-PSMA-617 worldwide. The large sample size and inclusion of nearly 3700 patients from 46 centers across France provide a comprehensive and representative overview of real-world practice. The diversity of participating sites reflects routine clinical settings. Furthermore, the inclusion of elderly and heavily pretreated patients enhances the applicability of the findings to the broader mCRPC population. The national multicenter design minimizes center-specific biases and supports the robustness of the results.

However, several limitations inherent to real-world data collection should be acknowledged. The declarative nature of data collection may have resulted in missing data. Furthermore, reliance on investigator-reported AEs could introduce reporting bias. Certain nonserious events, such as xerostomia, may not have been systematically reported by health care professionals, although they may have occurred. In addition, the absence of patient-reported outcome data represents a limitation, particularly for a treatment in which quality-of-life improvement is an important consideration. Another limitation relates to the evaluation criteria and imaging methods. In the EAP dataset, these data were left to the discretion of the treating centers, and therefore, these data were not collected.

Finally, a key limitation of this work is the reliance on between-study comparisons, specifically between real-world EAP data and the phase 3 VISION trial. Differences in patient selection, treatment exposure, and follow-up may limit the comparability of outcomes.

## CONCLUSION

The French EAP experience demonstrates that [^177^Lu]Lu-PSMA-617 maintains a favorable benefit–risk profile in a real-world mCRPC post-ARPI postchemotherapy population. These findings support its integration into routine clinical care and provide reassurance regarding its use in elderly and heavily pretreated patients or those with renal impairment. Our results also reveal a temporal trend toward the inclusion of patients with less advanced disease over time. Ongoing real-world subanalyses will help improve outcomes as clinical experience with [^177^Lu]Lu-PSMA-617 expands. The ongoing PSMAReach study (CAAA617A1FR03) is collecting harmonized real-world data from multiple centers across France (18). This retrospective analysis of patient records will provide complementary insights into treatment patterns, safety, and effectiveness, supporting optimized use of [^177^Lu]Lu-PSMA-617.

## DISCLOSURE

Funding was provided by Novartis, France. François Somme, Anne-Laure Giraudet, Yann Godbert, Sylvie Girault, Sophie Abadie-Lacourtoisie, Caroline Viala, Alix Douillet, and Stephanie Chêne receive financial support from Novartis. Clément Bailly receives support from Novartis, Boston Scientific, and Telix Pharmaceuticals. Lavinia Vija reports support from Novartis, Eisai, and Curium. Vincent Massard receives financial support from AstraZeneca, Novartis, Eli Lilly, Pfizer, Gilead Sciences, Bayer, Astellas Pharma, Johnson & Johnson, Menarini Stemline, Exact Sciences, Daiichi Sankyo, Astellas Pharma, MSD, Merck, and Roche. Philippe Barthélémy reports support from Astellas, Bristol-Myers Squibb, Ipsen, Eisai, MSD, Merck, Bayer, Johnson & Johnson, Astellas, Novartis, and Pfizer. Aude Fléchon receives support from Astellas, Amgen, Bayer, Johnson & Johnson, Novartis, Pfizer, and AstraZeneca. Guilhem Roubaud receives support from Astellas, AstraZeneca, Bayer, Janssen, Gilead, Merck, Novartis, and Pfizer. No other potential conflict of interest relevant to this article was reported.

## Notes

KEY POINTS **QUESTION:** What are the real-world efficacy and safety outcomes of [^177^Lu]Lu-PSMA-617 in the largest international cohort of patients with mCRPC, and how do they relate to the findings of the VISION trial? **PERTINENT FINDINGS:** [^177^Lu]Lu-PSMA-617 showed consistent efficacy and a manageable safety profile, even for elderly and heavily pretreated individuals, with outcomes similar to those in the VISION trial. Primary subanalyses confirmed benefits in elderly and retreated patients. **IMPLICATIONS FOR PATIENT CARE:** These real-world data support the use of [^177^Lu]Lu-PSMA-617 in routine care for a broad range of patients with mCRPC, including patients eligible for retreatment and those who may benefit from earlier use in the disease course.
